# Supplementary material for: Proteins Adsorbing onto Surface-Modified Nanoparticles: Effect of Surface Curvature, pH, and the Interplay of Polymers and Proteins Acid–Base Equilibrium
Source: Polymers (Basel). 2022 Feb 14;14(4):739. doi: 10.3390/polym14040739 (PMC8878797; doi:10.3390/polym14040739)
Supplement: Supplementary file 1 [file polymers-14-00739-s001.zip › polymers-1583828-supplementary.pdf]

Supplementary Materials: Proteins adsorbing onto surface modified nanoparticles: effect of surface curvature, pH and the interplay of polymers and proteins acid-base equilibrium

Estefania Gonzalez Solveyra [1], David H. Thompson [2] and Igal Szleifer [3,4,5*]

1. Theoretical Model

The theoretical approach employed in this work is a mean-field molecular theory that captures the coupling that exists between different physical and chemical interactions and explicitly includes molecular details on the system. The molecular theory is a density functional theory whose basic idea consists of writing the free energy of the system, as a functional of the probability distribution of the conformations of the end-tethered polymers and the proteins in solution, along with the spatial distribution of all molecular species in the system, including the tethered polymers, the solvent molecules, the ions and the proteins. In the following sections we present the formulation of the theory along with details on the numerical solutions and the molecular models used in our calculations.

1.1. Formulation of the theory

For a surface modified with a mixture of long and short polymers (which can be either neutral, acidic or basic) in contact with a solution containing different ions and a mixture of proteins, the Helmholtz free energy of the system has the following contributions:

$$F = \sum_{pol} \Big(-TS_{conf,pol} + F_{chem,pol} \Big) - TS_{mix} + \sum_{prot} \Big(-TS_{TR,prot} + E_{ads,prot} + F_{chem,prot} \Big) + E_{elect}, \tag{1}$$

where T is the temperature; $S_{conf,pol}$ is the conformational entropy of the tethered polymers chains and the index pol runs over the long and short polymers at the surface; S_{mix} corresponds to the mixing (translational) entropy of the small mobile species (water molecules, anions, cations); $S_{TR,prot}$ is the translational and rotational entropy of protein molecules; $E_{ads,prot}$ correspond to the adsorption energy of the proteins; $F_{chem,prot}$ and $F_{chem,pol}$ represent the free energy associated with protonation/deprotonation reactions of the titrable amino acids in the proteins. The index $prot$ runs over the different proteins in solution. Lastly, E_{elect} is the total electrostatic energy functional.

The conformational entropy of the grafted polymers per unit area is given by

$$-\frac{S_{conf}}{k_B A(R)} = \sum_{pol=l,s} \sigma_{pol} \sum_{\alpha} P_{pol}(\alpha) \ln P(\alpha). \tag{2}$$

where k_B is Boltzmann constant, the index pol accounts for the long (l) and short (s) polymers in the surface mixture, σ_{pol} is the surface density of the corresponding polymer type and $P_{pol}(\alpha)$ is the probability of finding a tethered polymer chain of type pol in conformation α. Each polymer conformation α is given by a set of the positions of all the monomers of the polymer chain type pol and it is an input to the theory. The probability distribution function (or pdf) is the central quantity in our theory, since any thermodynamic and structural properties of the polymers can be calculated from the probability distribution function.[1]

The next term in the free energy, $F_{chem,pol}$, accounts for the contribution arising from the acid-base equilibrium reactions of the tethered polymers (relevant when considering acidic or basic polymers on the surface). It includes the enthalpic and entropic cost associated with charging and uncharging of the titrable monomers:

$$-\frac{\beta F_{chem,pol}}{A(R)} = \int \mathrm{d}r\, G(r) \langle \rho_{pol}(r) \rangle \Big\{ f_{pol,p}(r) \Big[\ln f_{pol,p}(r) + \beta \mu^{\ominus}_{pol,p} \Big] + (1 - f_{pol,p}(r)) \Big[\ln(1 - f_{pol,p}(r)) + \beta \mu^{\ominus}_{pol,d} \Big] \Big\}. \quad (3)$$

The variable r is the coordinate that measures the distance from the tethering surface. We employed cartesian, cylindrical and spherical coordinates to reflect the symmetry of the planar surface, a cylindrical and a spherical nanoparticle respectively. We assumed the system to be laterally homogeneous and only explicitly anisotropic in the direction perpendicular to the surface, r. The function $G(r) = A(r)/A(R)$ describes the change in volume as a function of the distance away from the surface.[2] For planar systems, $G(r) = 1\ \forall r$, while in cylindrical and spherical coordinates it equals (r/R) or $(r/R)^2$, respectively. [3]

The local average (number) density of the polymers at position r is given by

$$\langle \rho_{pol}(r) \rangle = \frac{\sigma_{pol}}{G(r)} \sum_{\alpha} P_{pol}(\alpha) n_{pol}(\alpha; r). \quad (4)$$

Here $n_{pol}(\alpha; r)\mathrm{d}r$ is the number of segments that a polymer chain of type pol in conformation α has within volume element $[r, r + dr]$. The variable $n_{pol}(\alpha; r)$ is input to the molecular theory employed and depends on the molecular architecture and chemistry of each type of polymer.

Lastly in equation 3, $f_{pol,p}(r)$ corresponds to the local degree of protonation while the terms $\mu^{\ominus}_{pol,p}$ and $\mu^{\ominus}_{pol,d}$ correspond to the standard chemical potential of the protonated ("p") and deprotonated ("d") state, respectively. It is important to stress that the chemical state of the titrable monomers (either protonated of deprotonated) is not imposed but rather it is obtained as a result form the minimization of the free energy.

The next term in equation 1 corresponds to the translational (or mixing) entropy of all small mobile species in the system:

$$-\frac{S_{mix}}{k_B A(R)} = \sum_{\substack{k \in \{w, OH^-, H^+, \\ Na^+, Cl^-\}}} \int \mathrm{d}r\, G(r)\, \rho_k(r) \Big[\ln \rho_k(r) v_k - 1 + \beta \mu^{\ominus}_k \Big]. \quad (5)$$

The variable $\rho_k(r)$ is the local number density of mobile species k, v_k corresponds to its molecular (or ionic) volume (see Table S3 below), and $\mu^{\ominus}_k$ to its standard chemical potential. The standard chemical potential of water and ions in the system are related to the self-ionization of water.[1]

Next in the free energy functional is the contribution from the translational and rotational degrees of freedom of the protein molecules in solution:

$$-\frac{S_{TR_{prot}}}{k_B A(R)} = \int \mathrm{d}r\, G(r) \sum_{\theta_i} \rho_{prot}(\theta_i, r) \Big[\ln \rho_{prot}(\theta_i, r) V_{prot,tot} - 1 + \beta \mu^{\ominus}_{prot} \Big]. \quad (6)$$

where the subindex θ_i runs over the rotational conformations of protein i in solution ($i \in \{lys, GFP\}$, see below). $\rho_{prot}(\theta_i, r)$ is the local density of protein i when it is in conformation θ_i and $\mu^{\ominus}_{prot}$ corresponds to its standard chemical potential.

Next in the free energy is the interaction between the proteins and the surface, which drives the adsorption on the surface:

$$-\frac{\beta E_{ads,prot}}{A(R)} = \int \mathrm{d}r\, G(r) \sum_{\theta_i} \rho_{prot}(\theta_i, r)\, \beta U_{ps}(r). \quad (7)$$

where $U_{ps}(r)$ is the bare surface-protein potential, where r is the distance of the closest point of the protein to the surface. We considered the same potential for all the conformations of the proteins in the system.

The sixth term in the free energy, $F_{chem,prot}$, describes the free energy contribution arising from the acid-base equilibrium reactions for the titrable amino acids in the proteins:

$$-\frac{\beta F_{chem,prot}}{A(R)} = \int \mathrm{d}r\, G(r) \sum_{aat} \langle n_{aat}(r)\rangle \Big\{ f_{aat,p}(r)\Big[\ln f_{aat,p}(r) + \beta\mu^{\ominus}_{aat,p}\Big] + (1 - f_{aat,p}(r))\Big[\ln(1 - f_{aat,p}(r)) + \beta\mu^{\ominus}_{aat,d}\Big]\Big\}. \quad (8)$$

where the index aat runs over the titrable amino acids of the proteins (see Table S2 below), $f_{aat,p}(r)$ is the local fraction of protonated amino acid of type aat, and $\mu^{\ominus}_{aat,p}$ and $\mu^{\ominus}_{aat,d}$ are the standard chemical potentials of type aa amino acid residues that are protonated ("p") or deprotonated ("d"), respectively. Here again it is important to stress that the chemical state of the titrable amino acids is obtained as a result form the minimization of the free energy and it is not imposed as an input to the theory.

The ensemble average of the local density of residues is given by

$$\langle n_{aa}(r)\rangle = \int \mathrm{d}r' \,\frac{G(r')}{G(r)} \sum_{\theta_i} \rho(\theta_i, r')\; m_{aa}(\theta_i, r, r'). \quad (9)$$

where the subindex aa corresponds to any type of amino acid (titrable or neutral) of protein i, while $m_{aa}(\theta_i, r, r')$ refers to the number density of residues type aa that are found at r' when the center of mass of protein i in conformation θ_i is at position r. For each protein, the set $m_{aa}(\theta_i, r, r')$ is an input.

The last term in the free energy functional describes the electrostatic contribution and is given by:

$$\frac{\beta F_{elect}}{A(R)} = \beta \int \mathrm{d}r\, G(r)\Big[\langle \rho_q(r)\rangle \psi(r) - \frac{1}{2}\epsilon_0\epsilon_w (\nabla_r \psi(r))^2\Big]. \quad (10)$$

Here $\psi(r)$ is the electrostatic potential, $\langle \rho_q(r)\rangle$ corresponds to the total charge density, while ϵ_0 and ϵ_w refer to the dielectric permittivity of vacuum and the relative dielectric constant of the aqueous solution respectively, which is taken to be equal to that of water, $\epsilon_w = 78.5$. The total charge density $\langle \rho_q(r)\rangle$ is the sum of the charge number density of all charged species in the system:

$$\langle \rho_q(r)\rangle = \sum_{\substack{k\in\{Na^+,Cl^-,\\ H^+,OH^-\}}} e\, z_k\, \rho_k(r) + \sum_{pol} \langle \rho_{pol}(r)\rangle q_{pol}(r)\, e\, z_{pol} + \sum_{aat^i} \langle n_{aat^i}(r)\rangle q_{aat^i}(r)\, e\, z_{aat^i}. \quad (11)$$

Here, e is the unit of charge. The first term accounts for the charges of all mobile ions in solutions, with z_k corresponding to their valency. The next two terms considers the charges arising from the protonation/deprotonation reactions of the titrable groups of the tethered polymers and the titrable amino acids in the proteins (either deprotonated or protonated for acidic or basic species, respectively). The parameters z_{pol} and z_{aat^i} correspond to the electric charge of the titrable species (monomers or amino acids). For the titrable amino acids in protein i, the degree of charge $q_{aat^i}(r)$ corresponds to the fraction of residues of type aat that are ionized at position r, and it is related to the degree of protonation $f_{aat,p}(r)$ by:

$$q_{aat^i}(r) = \begin{cases} 1 - f_{aat,p}(r) & \text{for acidic residues} \\ f_{aat,p}(r) & \text{for basic residues} \end{cases} \quad (12)$$

Similarly, for the titrable monomomers in the polymer of type pol, we can write:

$$q_{pol}(r) = \begin{cases} 1 - f_{pol,p}(r) & \text{for acidic groups} \\ f_{pol,p}(r) & \text{for basic groups} \end{cases} \tag{13}$$

The repulsive interactions in the theory are modeled as excluded volume interactions. The intra chain interactions are considered exactly during generation of the polymer conformations (see below), while the intermolecular excluded volume interactions are accounted for by assuming that the system is incompressible at each position r:

$$\sum_{pol} \langle \phi_{pol}(r) \rangle + \sum_{prot} \langle \phi_{prot}(r) \rangle + \sum_{\substack{k \in \{w, OH^-, H^+, \\ Na^+, Cl^-\}}} \phi_k(r) = 1. \tag{14}$$

For each position r, these volume constraints are enforced through the introduction of the Lagrange multipliers $\pi(r)$, which represent the osmotic pressures induced in the system due to the inhomogeneous composition of polymers, protein, solvent and ions.

The variable $\langle \phi_p(r) \rangle$ corresponds to polymer type pol volume fraction and it is expressed as the polymer density times the sum over the different monomer states (protonated or deprotonated, in case they are titrable) weighted with the volume of those states:

$$\langle \phi_{pol}(r) \rangle = \langle \rho_{pol}(r) \rangle \left(f_{pol,p}(r) v_{pol,p} + f_{pol,d}(r) v_{pol,d} \right). \tag{15}$$

Here $v_{pol,p}$ and $v_{pol,d}$ correspond to the volume of the protonated and deprotonated monomer respectively, which were taken to be equal to the monomer volume v_p (see Table S1). The polymer local density $\langle \rho_{pol}(r) \rangle$ is given by equation 4.

The volume fraction of each protein in the system, $\langle \phi_{prot}(r) \rangle$, is given by:

$$\langle \phi_{prot}(r) \rangle = \sum_{aa} \langle n_{aa}(r) \rangle v_{aa}. \tag{16}$$

Here, the sum runs over all the amino acids type in each protein in the system, v_{aa} corresponds to the volume of the amino acid (see protein model below) and the average local density of each type of residue $\langle n_{aa}(r) \rangle$ is given by equation 9.

Finally, the volume fraction of the small mobile species is given by $\phi_k(r) = \rho_k(r) v_k$, where v_k is the volume of species k.

1.2. Minimization of the free energy

The free energy of the system is minimized with respect to $P_{pol}(\alpha)$ for each type of polymer, $\rho_k(r)$ with $k \in \{w, OH^-, H^+, Na^+, Cl^-\}$, $\rho_{prot}(\theta_i, r)$ for each protein i in solution, $f_{aat,p}(r)$ for each titrable amino acid in the proteins (see Table S2 below), $f_{pol,p}(r)$ for the titrable monomers (see Table S1 below), and varied with respect to the electrostatic potential, $\psi(r)$. These minimizations are done under the constraint of incompressibility and the fact that the system is in contact with an aqueous solution of proteins, cations, anions, protons, and hydroxyl ions. Therefore, the proper thermodynamic potential is the semi-grand potential [2,4–6], given by:

$$\frac{\beta W}{A(R)} = \frac{\beta F}{A(R)} - \sum_{k\in\{w,NaCl,Cl^-,OH^-\}} \beta\mu_k^{bulk} \int \mathrm{d}r\, G(r)\rho_k(r)$$
$$- \sum_{prot} \int \mathrm{d}r\, G(r) \sum_{\theta} \rho_{prot}(\theta,r)\beta\mu_{prot}^{bulk}$$
$$-\beta\mu_{H^+} \int \mathrm{d}r\, G(r) \left(\rho_{H^+}(r) + \sum_{aat} \langle n_{aat}(r)\rangle f_{aat,p}(r) + \sum_{pol} \langle \rho_{pol}(r)\rangle f_{pol,p}(r) \right)$$
$$+ \int \mathrm{d}r\, G(r)\, \beta\pi(r) \left(\sum_{pol} \langle \phi_{pol}(r)\rangle + \sum_{prot} \langle \phi_{prot}(r)\rangle + \sum_{\substack{k\in\{w,OH^-,H^+,\\ Na^+,Cl^-\}}} \phi_k(r) - 1 \right). \tag{17}$$

where the first extra term corresponds to the chemical potential of ions in the bulk solution, the second one to the bulk chemical potential of the proteins, the third to the chemical potential of the protons in the system (taking into account the free protons in solution and those that are in the protonated states of the titrable species, amino acids and monomers), and the last one to the incompressibility constraint, enforced through the introduction of the Lagrange multipliers $\pi(r)$, which physical meaning was mentioned above.

The complete potential to minimize is given by:

$$\frac{\beta W}{A(R)} = \sum_{pol=l,s} \sigma_{pol} \sum_{\alpha} P_{pol}(\alpha) \ln P_{pol}(\alpha)$$
$$+ \int \mathrm{d}r\, G(r) \sum_{pol} \langle \rho_{pol}(r)\rangle \left\{ f_{pol,p}(r) \left[\ln f_{pol,p}(r) + \beta\mu_{pol,p}^{\ominus} \right] \right\}$$
$$+ \int \mathrm{d}r\, G(r) \sum_{pol} \langle \rho_{pol}(r)\rangle \left\{ (1 - f_{pol,p}(r)) \left[\ln(1 - f_{pol,p}(r)) + \beta\mu_{pol,d}^{\ominus} \right] \right\}$$
$$+ \int \mathrm{d}r\, G(r) \sum_{\theta_i} \rho_{prot}(\theta_i,r) \left[\ln \rho_{prot}(\theta_i,r) V_{prot,tot} - 1 + \beta\mu_{prot}^{\ominus} + \beta U_{ps}(r) - \beta\mu_{prot}^{bulk} \right]$$
$$+ \int \mathrm{d}r\, G(r) \sum_{aat} \langle n_{aat}(r)\rangle \left\{ f_{aat,p}(r) \left[\ln f_{aat,p}(r) + \beta\mu_{aat,p}^{\ominus} \right] \right\}$$
$$+ \int \mathrm{d}r\, G(r) \sum_{aat} \langle n_{aat}(r)\rangle \left\{ (1 - f_{aat,p}(r)) \left[\ln(1 - f_{aat,p}(r)) + \beta\mu_{aat,d}^{\ominus} \right] \right\}$$
$$+ \sum_{\substack{k\in\{w,OH^-,H^+,\\ Na^+,Cl^-\}}} \int \mathrm{d}r\, G(r)\, \rho_k(r) \left[\ln \rho_k(r) v_k - 1 + \beta\mu_k^{\ominus} - \beta\mu_k^{bulk} \right]$$
$$+ \int \mathrm{d}r\, G(r) \left[\langle \rho_q(r)\rangle\, \beta\psi(r) - \frac{1}{2}\epsilon_0\epsilon_w (\nabla_r \psi(r))^2 \right]$$
$$+ \int \mathrm{d}r\, G(r)\, \beta\pi(r) \left(\sum_{pol} \langle \phi_{pol}(r)\rangle + \sum_{prot} \langle \phi_{prot}(r)\rangle + \sum_{\substack{k\in\{w,OH^-,H^+,\\ Na^+,Cl^-\}}} \phi_k(r) - 1 \right)$$
$$- \int \mathrm{d}r\, G(r)\, \beta\mu_{H^+} \left(\rho_{H^+}(r) + \sum_{aat} \langle n_{aat}(r)\rangle f_{aat,p}(r) + \sum_{pol} \langle \rho_{pol}(r)\rangle f_{pol,p}(r) \right) \tag{18}$$

Minimization of the thermodynamic potential in 18 leads to the following expression for the local volume fraction of the solvent:

$$\phi_w(r) = \rho_w(r) v_w = \exp(-\beta\pi(r) v_w), \tag{19}$$

while for the local density of the ions we obtain:

$$\rho_k(r) = \frac{1}{v_k} \exp\Big(\beta\mu_k^{bulk} - \beta\mu_k^{\ominus} - \beta\pi(r) v_k - \beta\psi(r) z_k e\Big). \tag{20}$$

It is important to point out that the chemical potential of water does not need to be specified explicitly, because the incompressibility constraint reduces the number of thermodynamic independent variables. In this way, the chemical potentials, μ_k, actually correspond to exchange chemical potentials, which we define as the difference between the chemical potential of the species k and that of water.[2] The values of the exchange chemical potential of the remaining species can be expressed by relating them to their bulk concentrations: $\rho_k^{bulk} v_k = \exp(-\beta\mu_k^{\ominus} + \beta\mu_k^{bulk} - \beta\pi^{bulk} v_k - \beta\psi^{bulk} z_k)$, with $\psi^{bulk} = 0$, see below at Eq. 21.

Functional variation of the free energy with respect to the electrostatic potential yields the Poisson equation and its boundary conditions:

$$-\epsilon_0\epsilon_w \nabla_r^2 \psi(r) = \langle \rho_q(r) \rangle; \quad -\epsilon_0\epsilon_w \frac{d\psi(r)}{dr}\bigg|_{r=R} = \sigma_q; \quad \lim_{r\to\infty} \psi(r) = 0. \tag{21}$$

in order to ensure the global charge-neutrality constraint of the system in equilibrium conditions:

$$\int \mathrm{d}r\, G(r)\, \langle \rho_q(r) \rangle = 0. \tag{22}$$

Minimization with respect to $P_{pol}(\alpha)$ yields the probability distribution function (pdf) for each type of polymer:

$$P_{pol}(\alpha) = \frac{1}{Z_{pol}} \exp\Big\{ -\int \mathrm{d}r\, n_{pol}(\alpha; r) \quad [\quad \beta\pi(r) v_{pol} + \beta\psi(r) z_{pol} e + \ln q_{pol}(r) + \beta\mu_{pol,d}^{\ominus}] \Big\} \tag{23}$$

where the normalization constant

$$Z_{pol} = \sum_\alpha \exp\Big\{ -\int \mathrm{d}r\, n_{pol}(\alpha; r) \quad [\quad \beta\pi(r) v_{pol} + \beta\psi(r) z_{pol} e + \ln f_{pol,d}(r) + \beta\mu_{pol,d}^{\ominus}] \Big\} \tag{24}$$

ensures that for each type of polymer, $\sum_\alpha P_{pol}(\alpha) = 1$. For further details on is referred to the supporting material of Refs. [1,2] .

Minimization with respect to the polymer protonation fraction, $f_{pol,p}(r)$, results in the following expression:

$$\frac{1 - f_{pol,p}(r)}{f_{pol,p}(r)} = K_{a,pol}^{\ominus} \frac{\rho_{H^+}(r)}{\rho_w(r)} \tag{25}$$

The variable $K_{a,pol}^{\ominus} = \exp(-\beta\Delta G_{pol}^{\ominus})$ corresponds to the thermodynamic acid-base equilibrium constant and $\Delta G_{pol}^{\ominus}$ is the standard free energy change given by $\Delta G_{pol}^{\ominus} = \mu_{pol,d}^{\ominus} + \mu_{H^+}^{\ominus} - \mu_{porl,p}^{\ominus}$. The chemical equilibrium constant $K_{pol}^{\ominus}$ is related to the experimental equilibrium constant $K_{a,pol} = C \exp(-\beta\Delta G_{pol}^{\ominus})$ of a single acidic monomer in infinitely dilute solution. The constant C is required for consistency of units and equal to $C = 1/N_A v_w$, where N_A is Avogadro's number.

Following 13, the degree of charge of the titrable monomers is related to expression 25:

$$\frac{q_{pol}(r)}{1-q_{pol}(r)} = \left(K^{\ominus}_{a,pol}\frac{\rho_{H^+}(r)}{\rho_w(r)}\right)^{\pm 1} \tag{26}$$

where the positive and negative signs correspond to acidic or basic monomers, respectively.

Minimization with respect to $\rho_{prot}(\theta_i, r)$ yields the following expression for the local density of protein i in conformation θ_i:

$$\begin{aligned} \rho_{prot}(\theta_i, r)V_{prot} = {} & \exp\left(\beta\mu^{bulk}_{prot} - \beta\mu^{\ominus}_{prot}\right) \\ & \times \exp\left(\beta U_{ps}(r)\right) \\ & \times \exp\left\{ -\int dr' \sum_{aa} m_{aa}(\theta_i, r, r')\ \beta\pi(r')v_{aa} \right\} \\ & \times \exp\left\{ -\int dr' \sum_{aat} m_{aat}(\theta_i, r, r')\ \left[\ln q_{aat}(r') + \beta\psi(r')z_{aat}\right] \right\} \\ & \times \exp\left\{ -\sum_{aat,a} cn_{aat}\ \beta\mu^{\ominus}_{aat,d} - \sum_{aat,b} cn_{aat}\ \left[\beta\mu^{\ominus}_{aat,p} - \beta\mu_{H^+}\right] \right\} \end{aligned} \tag{27}$$

The first term in 27 can be determined from the composition of the bulk solution in contact with the surface (see next section). The sub index aa runs over all the amino acids in protein i, while aat only counts the titrable ones. In the last line, cn_{aat} corresponds to the total count of acidic or basic titrable amino acids, the first term running over the acidic residues (aat, a), while to the second one does so over basic amino acids (aat, b).

Lastly, minimization of the thermodynamic potential with respect to the degree of protonation of each titrable amino acids in the proteins, $f_{aat,p}(r)$, yields identical expressions to equation 25, leading to the analogue expressions for $q_{aat}(r)$ as in 26.

Note that in Eq. (19) through Eq. (27) the unknowns are the Lagrange multipliers or lateral pressures, $\pi(r)$, and the electrostatic potential, $\psi(r)$. All the other quantities can be expressed as a function of $\psi(r)$ and $\pi(r)$. The solutions of those variables can be obtained numerically, by replacing the expressions of the volume fractions and charge density of all components into the incompressibility constraint (Eq. 14) and the Poisson equation (Eq. 21). This results in a set of non-linear integro differential equations whose solution will determine $\psi(r)$ and $\pi(r)$. Resorting to a discretization scheme, the differential equations are converted into a set of coupled non-linear algebraic equations that can be solved by standard numerical techniques. [7] Further below we present details on the discretization procedure and numerical methods.

The inputs required to solve the non-linear equations are a set of polymer and protein conformations, the bulk solution conditions (pH, salt and proteins concentrations), the volume of all species in the system, the surface density of the polymers end-tethered to the nanoparticle, the morphology and curvature of the nanoparticle, and the various acid-base equilibrium constants pKa. In the "Molecular Models" section we present details on the chain and protein models used to generate a set of macromolecular conformations, along with the relevant properties of other species in solution.

1.3. Bulk solution

The composition of the bulk solution in equilibrium with the end-tethered polymer surface determines the chemical potential of mobile species in solution (ions, solvent, proteins), as reflected in Eq. 17. In this section we derive the bulk expressions of densities and charge fractions by taking the limit of $r \to \infty$ in the expression of the ions local densities (Eq. 20), the charged fraction of titrable amino acids in the proteins (Eq. 26) and the local density of proteins (Eq. 27).

The bulk density of ions in solution has the following expression:

$$\rho_k^{bulk} v_k = \phi_k^{bulk} = \exp(\beta\mu_k^{\ominus} - \beta\mu_k - \beta\pi^{bulk} v_k - \beta\psi^{bulk} z_k) \tag{28}$$

with $\psi_{bulk} = 0$ (see Eq. 21) and $k \in \{H^+, OH^-, Na^+, Cl^-\}$.
For the density of proteins we get:

$$\begin{aligned} \rho_{prot}^{bulk}(\theta_i) V_{prot} &= \exp\left(\beta\mu_{prot}^{bulk} - \beta\mu_{prot}^{\ominus}\right) \\ &\times \exp\left\{ -\sum_{aa} cn_{aa}\, \beta\pi^{bulk} v_{aa} \right\} \\ &\times \exp\left\{ -\sum_{aat} cn_{aat} \left[\ln q_{aat}^{bulk} + \beta\psi^{bulk} z_{aat}\right] \right\} \end{aligned} \tag{29, 30}$$

where cn corresponds to the count of amino acids of type aa, that runs over all amino acids in the protein i, or just the titrable ones aat.

For the titrable amino acids in the proteins (see Table S2 below), their bulk degree of charge is given by:

$$\frac{q_{aat}^{bulk}}{1 - q_{aat}^{bulk}} = \left(K_{a,aat}^{\ominus} \frac{\rho_{H^+}^{bulk}}{\rho_w^{bulk}}\right)^{\pm 1} \tag{31}$$

where the positive and negative signs correspond to acidic or basic monomers, respectively.

The electrostatic potential in the bulk solution, ψ^{bulk} has a constant value that is an input to the theory and it is set $\psi^{bulk} = 0$.

The system in the bulk solution is also assumed to be incompressible, leading to:

$$\sum_{prot} \langle \phi_{prot}^{bulk} \rangle + \sum_{\substack{k \in \{w, OH^-, H^+, \\ Na^+, Cl^-\}}} \phi_k^{bulk} = 1. \tag{32}$$

The only unknown to the determine the bulk densities and charged fractions of amino acids is the osmotic pressure π^{bulk}, which is computed by replacing expressions 28, 29 and 29 in the bulk incompressibility constraint above (32) and solving numerically the equation for each given value of bulk pH, salt and protein concentrations (which are input for our calculations). Once π^{bulk} is computed, the terms containing bulk and reference chemical potentials in equations 28 and 29 can be replaced in equations 20 and 27 above.

1.4. Discretization and Numerical methodology

Numerical solutions for the lateral pressure, $\pi(r)$ and the electrostatic potential, $\psi(r)$ are obtained by discretizing the packing constraint (Eq. 14) and the generalized Poisson equation (Eq. 21). This is done by dividing the r-coordinate into M_r cartesian, cylindrical or spherical shells of thickness δ (for a planar, cylindrical or spherical surface respectively). We used a discretization length $\delta = 0.5$ nm. All the position dependent functions derived above are assumed to be constant within a given layer, such that integrations can be replaced by summations. In this way, the integral of a general position dependent function $f(r)$ becomes:

$$\int \mathrm{d}r\, G(r) f(r) = \sum_i \int_{(i-1)\delta+R}^{i\delta+R} \mathrm{d}r\, G(r) f(r) \approx \delta \sum_i f(i) \Delta G(i), \tag{33}$$

with

$$\Delta G(i) = \frac{1}{\delta} \int_{(i-1)\delta+R}^{i\delta+R} \mathrm{d}r\, G(r). \tag{34}$$

where $f(i)$ corresponds to the value which the function $f(r)$ attains within the layer located between $(i-1)\delta + R \leq r < i\delta + R$ (for spherical and cylindrical nanoparticles, R corresponds to the radius, while for planar surfaces $R = 0$). As mentioned at the beginning of this section, $G(r) = A(r)/A(R)$ describes the change in volume as a function of the distance away from the surface and it depends on its geometry.[3]

The discretized densities of water (19) and ions (20) in solution read:

$$\phi_w(i) = \rho_w(i)v_w = \exp(-\beta\pi(i)v_w), \quad (35)$$
$$\phi_k(i) = \phi_k^{bulk} \exp\left[-\beta(\pi(i) - \pi^{bulk})v_k - z_k\beta\psi(i)\right]. \quad (36)$$

where $\phi_k(i)$ is given by equation 28 and is input to the theory for each species k.

The discretized form of the pdf of the polymers (23) and the polymer density (4) are:

$$P_{pol}(\alpha) = \frac{1}{Z_{pol}} \prod_{i=1}^{M_r} \exp\left[-n_{pol}(\alpha;i)\Big(\beta\pi(i)v_{pol} + e\beta\psi(i) + \ln(q_{pol}(i))\Big)\right] \quad (37)$$
$$\langle\rho_{pol}(i)\rangle = \frac{\sigma}{\delta\Delta G(i)} \sum_{\alpha} P_{pol}(\alpha)n_{pol}(\alpha;i) \quad (38)$$
$$n_{pol}(\alpha;i) \equiv \int_{(i-1)\delta+R}^{i\delta+R} \mathrm{d}r\, n_{pol}(\alpha;r). \quad (39)$$

For the proteins in the system, the discretized forms of the volume fractions (16), local density (27) and average local density of each type of residue (9):

$$\langle\phi_{prot}(i)\rangle = \sum_{aa} \langle n_{aa}(i)\rangle v_{aa} \quad (40)$$
$$\langle n_{aa}(i)\rangle = \sum_{j=1}^{M_r} \sum_{\theta_{prot}} \frac{\Delta G(j)\, \rho_{prot}(\theta,j) m_{aa}(\theta,i,j)}{G(r)} \quad (41)$$
$$\begin{aligned}\rho_{prot}(\theta,j) = {} & \rho_{prot}^{bulk}(\theta) \times \exp\left(\beta\pi^{bulk}V_{prot}\right) \times \exp\Big(\sum_{aat} cn_{aat}\ \ln q_{aat}^{bulk}\Big) \\ & \times \exp\big(\beta U_{ps}(j)\big) \\ & \times \exp\Big\{-\sum_{h=1}^{M_r} \beta\pi(h) \sum_{aa} m_{aa}(\theta,j,h)\ v_{aa}\Big\} \\ & \times \exp\Big\{-\sum_{h=1}^{M_r} \sum_{aat} m_{aat}(\theta,j,h)\ \left[\ln q_{aat}(h) + \beta\psi(h)z_{aat}\right]\Big\}.\end{aligned} \quad (42)$$

Following equation 26, the discretized expressions for the charged fractions of titrable species in the system are:

$$q_\gamma(i) = \frac{1}{1 + \left(K_{a,\gamma}^{\ominus} \frac{\rho_{H^+}(i)}{\rho_w(i)}\right)^{\pm 1}} \quad (43)$$

where γ corresponds to the titrable monomers in the polymers and the titrable amino acids in the proteins. Their acidity equilibrium constants are detailed in Tables S2 and S1. The positive and negative signs in the denominator correspond to acidic or basic residues, respectively.

The discrete local density of charges (11) has the following form:

$$\langle\rho_q(i)\rangle = \sum_{\substack{k\in\{Na^+,Cl^-,\\ H^+,OH^-\}}} e\,z_k\,\rho_k(i) + \sum_{pol}\langle\rho_{pol}(i)\,\rangle q_{pol}(i)\,e\,z_{pol} + \sum_{aat^i}\langle n_{aat^i}(i)\,\rangle q_{aat^i}(i)\,e\,z_{aat^i}. \quad (44)$$

For grid cell $i = 1...M_r$, the packing constraint (14) in discrete form reads:

$$\sum_{pol}\langle\phi_{pol}(i)\rangle + \sum_{prot}\langle\phi_{prot}(i)\rangle + \sum_{\substack{k\in\{w,OH^-,H^+,\\ Na^+,Cl^-\}}} \phi_k(i) = 1. \quad (45)$$

Finally, the discretized Poisson equation (21) takes different forms, depending on the system's geometry.

In cartesian coordinates (planar surface) we have:

$$\psi(i+1) - 2\psi(i) + \psi(i-1) = -\epsilon_w\epsilon_0\,\delta^2\,\langle\rho_q(i)\rangle, \quad (46)$$

For cylindrical coordinates:

$$\left(1+\frac{\delta}{r_i}\right)\psi(i+1) - 2\psi(i) + \left(1-\frac{\delta}{r_i}\right)\psi(i-1) = -\epsilon_w\epsilon_0\,\delta^2\,\langle\rho_q(i)\rangle, \quad (47)$$

where $r_i = [(i-1/2)\delta + R]$ denotes the middle of the cylindrical layer (i) in the radial direction r.

For spherical coordinates:

$$\left(1+\frac{\delta}{2r_i}\right)\psi(i+1) - 2\psi(i) + \left(1-\frac{\delta}{2r_i}\right)\psi(i-1) = -\epsilon_w\epsilon_0\,\delta^2\,\langle\rho_q(i)\rangle, \quad (48)$$

where $r_i = [(i-1/2)\delta + R]$ denotes the middle of the spherical layer (i) in the radial direction r.

The discretized electrostatic boundary conditions are:

$$\psi(1) - \psi^{surf} = 0 \quad (49)$$
$$\psi^{bulk} = \psi(M_r) = 0. \quad (50)$$

where ψ^{surf} is the electrostatic potential at the surface: $\psi^{surf} = \psi(R)$ for spherical or cylindrical nanoparticles of radius R, whereas for planar surfaces we have $\psi^{surf} = \psi(0)$. Far away from the surface, the electrostatic potential, ψ^{bulk}, vanishes.

For a given set of bulk solution details (pH, salt and proteins concentration) and surface conditions (surface morphology and curvature, surface density of each polymer, and chemical details of the polymers), the only unknowns in the previous equations are the discretized lateral pressures ($\pi(i)$) and electrostatic potential ($\psi(i)$). Their values can be obtained by solving the set of coupled nonlinear equations given by equations 45 and the appropriate version of the discretized Poisson equation (46, 47, 48), using standard numerical methods. [7]

2. Molecular models

2.1. Grafted Polymers

The surface in contact with the proteins solution was modified with a mixture of short (s) and long polymer (l). In order to solve the equations derived above, we need to generate a large and representative set of conformations for each type of polymer. To do so, we use the three-state rotational isomeric state (RIS) model. [8] The conformations are generated by a Monte Carlo sampling procedure that takes into account the self-avoidance of the polymer chain. Then, through appropriate translational and rotational adjustments, the non-intersecting generated chain conformations are end-tethered the surface of the NP of

given geometry and radius. Internal self-avoidance of the chain and the chain with the NP core are enforced. The used parameters of monomer volume and segment length are presented in Table S1. The set of chain conformations for each type of polymer (long and short) is generated once for each sequence and is used for all the calculations reported in this paper. The number of conformations is $1 \cdot 10^6$ for each type of polymer.

The surface mixture is characterized by two input parameters, σ_{pol} and x_l, that correspond to the total polymer surface density ($\sigma_{pol} = \sigma_l + \sigma_s$) and the fraction of long polymers on the surface respectively ($x_l = \sigma_l/\sigma_{tot}$, $x_l \in [0, 1]$).

The monomers in the short polymers were considered neutral, while the for the long one three possible types were considered: neutral, acidic or basic. Long polymers have 45 monomers, while short ones have 10. The choice of polymer size follows the commonly commercialized PEG products with molecular weights of 2000 and 500, respectively. The monomer volume was considered the same in all types of polymers and equal to that of PEG, as done in previous calculations.[9] The characteristics of each type of monomer (titrable or neutral) are listed in Table S1.

Table S1. Volume, pKa and charge of the deprotonated or protonated state for acidic or basic monomers, respectively.

Monomer type	v_p $[nm^3]$	pKa	q $[e]$
Neutral	0.065	-	0
Acidic	0.065	5.0	-1
Basic	0.065	9.0	+1

2.2. Protein model

We considered the modified surfaces in contact with a solution containing a protein mixture composed of lysozyme and Green Fluorescent Protein (GFP). Following a coarse graining scheme similar to Refs. [5,6], proteins were modeled in a coarse grain scale in which each amino acid in the proteins is represented by a single solid bead centered at the position of the corresponding α-carbon, as depicted in the left panel of Figure S1. The position and sequence of all atoms in the proteins are taken from the crystallographic structure PDB files (193L[10] and 1EMA[11] for lyzozyme and GFP respectively). The relative position of all beads remains frozen to the initial structure of the PDB structure, irrespective of solution conditions. This means that we did not include changes in their configurations neither in bulk solution nor when they are in contact with the surface. In this way, the proteins are modeled as rigid bodies, while retaining full translational and rotational degrees of freedom. Lysozyme is known undergo negligible conformational changes upon adsorption,[12,13] while the β-barrel of GFP is known to be stable and rigid.[14] Hence in our model, we do not take into account conformational changes upon protein adsorption on the NP surface.

The volume of each coarse grain bead is taken as the molecular volume of the amino acid, which was computed using the package VOIDOO[15]. Amino acids are considered hydrophilic and are classified either as neutral or titrable. Among the latter, aspartic acid (ASP), glutamic acid (GLU), and tyrosine (TYR) are considered acidic groups, while arginine (ARG), histidine (HIS), and lysine (LYS) are basic. These properties of the amino acids are summarized in Figure S1, right panel. Each titrable bead is characterized by an intrinsic acidic constant, while all other amino acids are considered charge neutral. The pKa values for the titrable amino acids correspond to experimental values averaged over different proteins and are summarized in Table S2.[16]

2.3. Other species in solution

Regarding the small species in solution (ions and water molecules), we used the volumes and charges listed in Table S3.

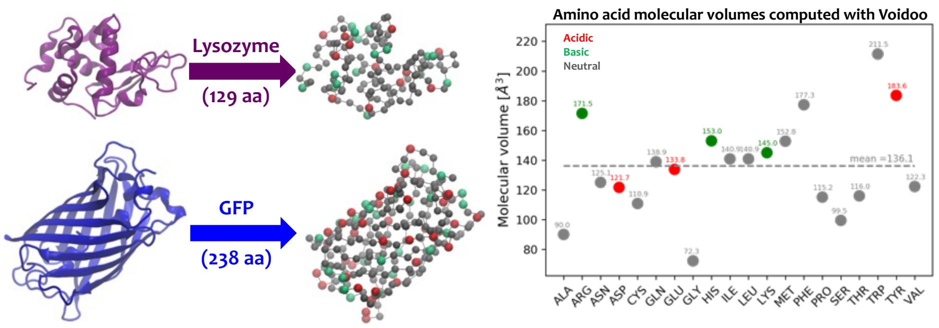

Figure S1. (left panel) Schematic representation of the coarse grain protein model: each amino acid is represented by a single solid bead centered at the position of the corresponding α-carbon provided in the crystallographic structure PDB file. (right panel) Amino acids molecular volumes computed with VOIDOO.

Table S2. Acid-base reaction constants (pKa) and count (cn) of each residue type in lysozyme and GFP for titrable amino acids.

	ASP	GLU	TYR	ARG	HIS	LYS
pKa [16]	3.5	4.2	10.3	12.0	6.6	10.5
cn^{lys}	7	2	3	11	1	6
cn^{GFP}	17	15	9	7	9	19

Table S3. Volume and charge of small mobile species

	$v\ [nm^3]$	$q(e)$
w	0.03	0
H^+	0.03	+1
OH^-	0.03	-1
Na^+	0.0044	+1
Cl^-	0.011	-1

3. Additional Results

3.1. Single protein adsorption onto NPs

3.1.1. Effect of surface morphology for lysozyme adsorption

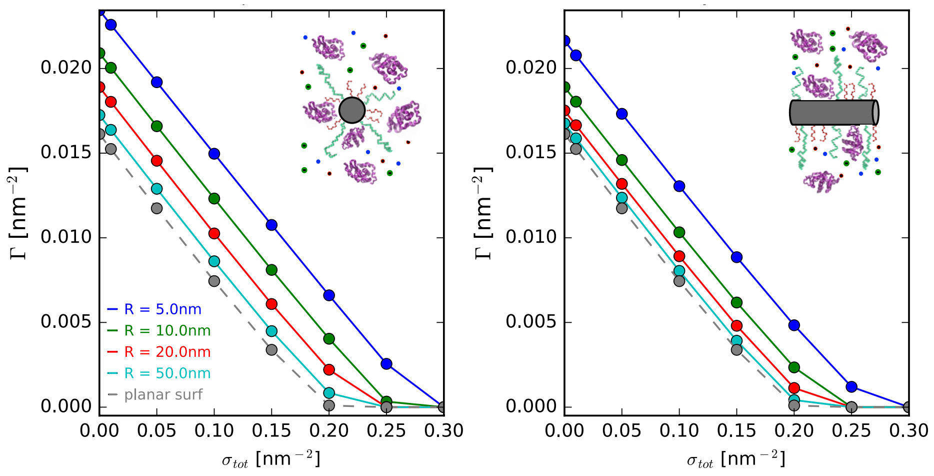

Figure S2. Adsorption isotherms of lysozyme onto NPs of different morphology and curvatures as a function of total polymer surface density (σ_{tot}). Long polymers are neutral and the surface composition is fixed, x_l^{surf} = 0.5. The bulk solution pH is 11, c_{salt} = 1mM, c_{lys} = 10^{-4} M. The radii of the spherical and cylindrical NP are indicated in the legend, as well as the limiting case for a planar surface.

3.1.2. Effect of surface composition for lysozyme adsorption

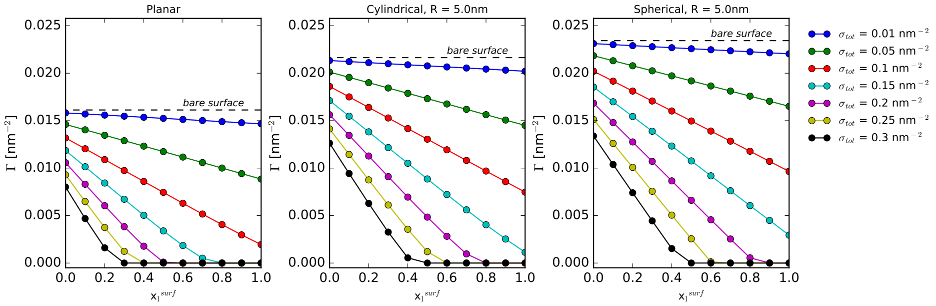

Figure S3. Adsorption isotherms of lysozyme onto NPs of different morphology and curvature as a function of the composition of the surface polymer mixture (x_l^{surf}), for different total polymer surface density (σ_{tot}), as indicated in the legend. For the curved systems, R = 5nm. Long polymers are neutral. The bulk solution pH is 11, c_{salt} = 1mM, c_{lys} = 10^{-4} M.

3.1.3. Molecular organization for lysozyme adsorption

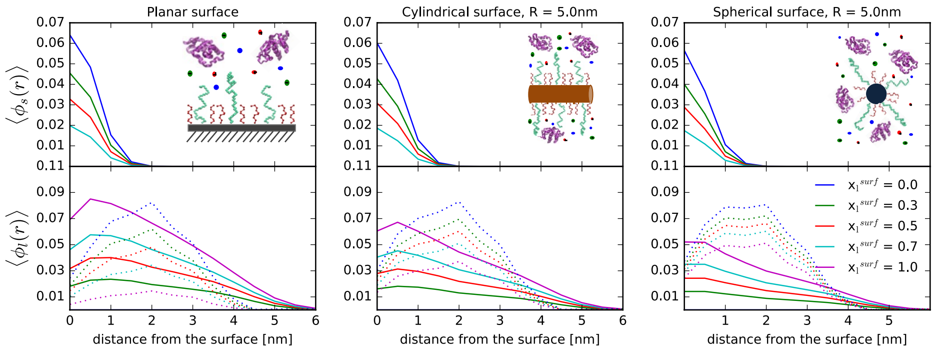

Figure S4. Volume fraction of the end-tethered short (upper panels) and long polymers (lower panels) as a function of the distance to the surface for planar, cylindrical and spherical NPs, as indicated in the figure headers. For the curved systems, R = 5nm. Long polymers are neutral, and the surface details are fixed: σ_{tot} = 0.1 nm^{-2} and x_l^{surf} = 0.5. The bulk solution pH is 11, c_{salt} = 1mM, c_{lys} = 10^{-4} M. Lines correspond to different surface compositions, as indicated in the legend. In the lower panels, the dotted lines correspond to the volume fraction of lysozyme.

3.1.4. Effect of surface composition for lysozyme adsorption

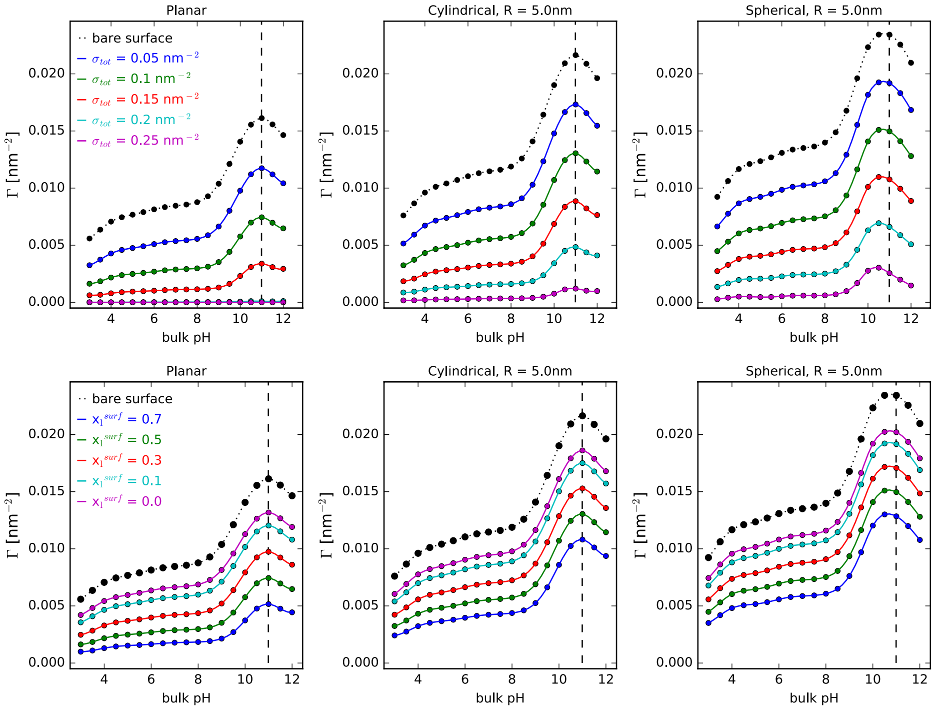

Figure S5. Lysozyme adsorption onto planar, cylindrical and spherical surfaces, as indicated in the header of each panel, as a function of bulk pH. Polymers are neutral. Effect of total polymer surface density for fixed surface composition (x_l^{surf} = 0.5, upper panels) and surface composition for fixed surface density (σ_{tot} = 0.1 nm^{-2} , lower panels), as indicated in the legends. The bulk solution conditions are c_{salt} = 1mM, and c_{lys} = 10^{-4} M.

3.1.5. Effect of surface curvature in NPs coated with weak polyelectrolytes for lysozyme adsorption

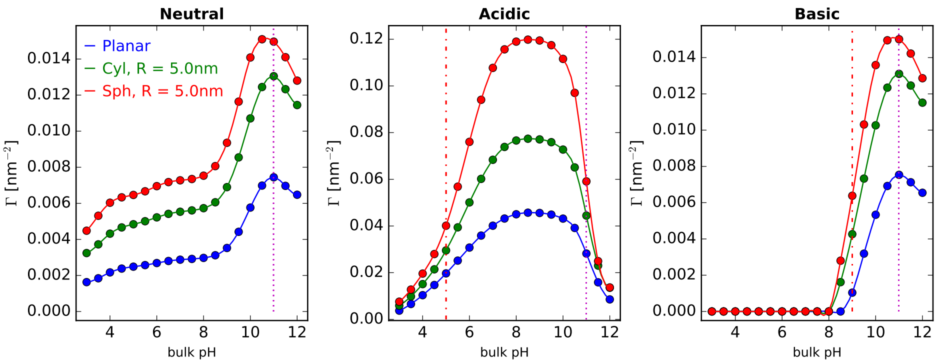

Figure S6. Lysozyme adsorption onto planar, cylindrical and spherical surfaces, as indicated in the legend, as a function of bulk pH. For the curved systems, R = 5nm. Long polymers are neutral, acidic or basic, as indicated in the the header of each panel. Surface details are fixed, σ_{tot} = 0.1 nm^{-2} and x_l^{surf} = 0.5. The bulk solution conditions are c_{salt} = 1mM, and c_{lys} = 10^{-4} M.

3.1.6. Effect of surface composition in NPs coated with weak polyelectrolytes for lysozyme adsorption

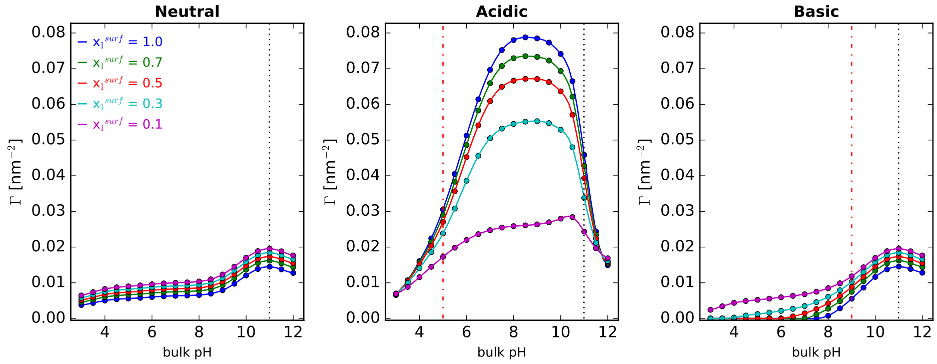

Figure S7. Effect of surface details on lysozyme adsorption onto cylindrical NPs, R = 5nm. Long polymers are neutral, acidic or basic, as indicated in the the header of each panel. The total polymer surface density is fixed, σ_{tot} = 0.1 nm^{-2}, while the composition of the surface mixture is varied, as indicated in the legend. The bulk solution conditions are c_{salt} = 1mM, and c_{lys} = 10^{-4} M. The dotted black lines correspond to the isoelectric point of lysozyme in dilute solution (pI=10.99), while the line-dot lines in the central and right panels correspond to the pKa values of the acidic and basic monomer (pKa^{acid} = 5.0, pKa^{basic} = 9.0).

3.1.7. Effect of surface details on GFP adsorption

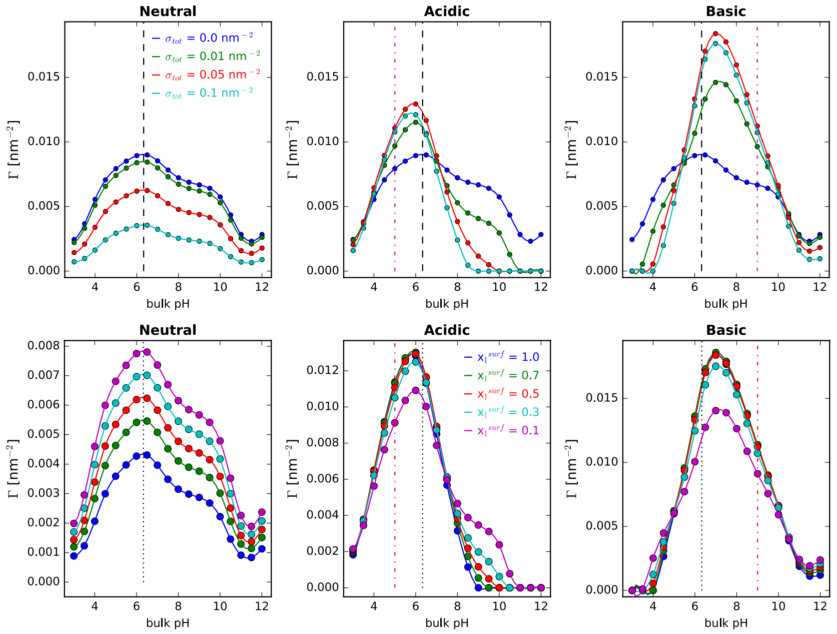

Figure S8. Effect of surface details on GFP adsorption onto cylindrical NPs, R = 5nm. Long polymers are neutral, acidic or basic, as indicated in the the header of each panel. (upper panels) The total polymer surface density is fixed, σ_{tot} = 0.1 nm^{-2}, while the composition of the surface mixture is varied, as indicated in the legend. (lower panels) The composition of the surface polymer mixture is fixed, x_l^{surf} = 0.5, while the total polymer surface density is varied, as indicated in the legend. For all panels, the bulk solution conditions are c_{salt} = 1mM, and c_{GFP} = 10^{-4} M. The dotted black lines correspond to the isoelectric point of lysozyme in dilute solution, while the line-dot lines in the central and right panels correspond to the pKa of the acidic and basic monomer respectively (pKa^{acid} = 5.0, pKa^{basic} = 9.0).

3.1.8. Local pH for GFP adsorption

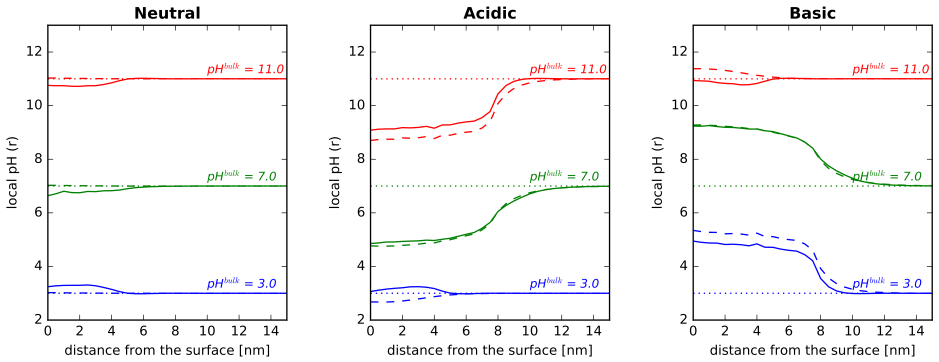

Figure S9. Local pH as a function of the distance to the surface for the adsorption of GFP onto cylindrical NPs, R=5nm. Long polymers are neutral, acidic or basic, as indicated in the the header of each panel. Surface details are fixed, σ_{tot} = 0.1 nm^{-2} and x_l^{surf} = 0.5. The bulk solution conditions are c_{salt} = 1mM, and c_{GFP} = 10^{-4} M, while the pH is changed , as indicated in the legends. Dotted lines correspond to the bulk pH value, while dot-dash lines correspond to the local pH without any protein in solution.

3.1.9. Acid-base eq. of titrable amino acids for GFP adsorption

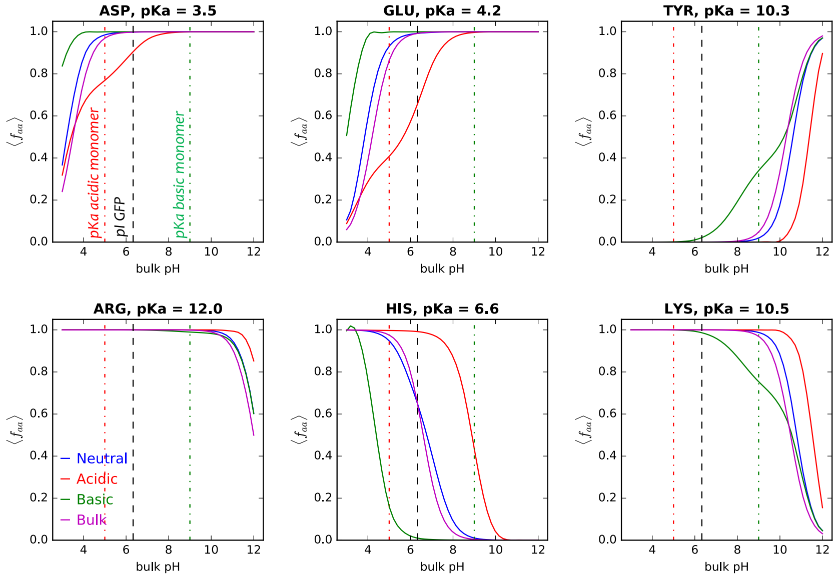

Figure S10. Average of the fraction of deprotonated (acidic, upper panels) or protonated (basic, lower panels) amino acids of adsorbed GFP onto cylindrical NPs (R=5nm) as a function of bulk pH. The amino acid name, type and pKa are indicated in the header of each panel. Surface details are fixed, σ_{tot} = 0.1 nm^{-2} and x_l^{surf} = 0.5. The bulk solution conditions are c_{salt} = 1mM, and c_{GFP} = 10^{-4} M. Blue, red, and green full lines correspond to NPs grafted with neutral, acidic and basic polymers, as indicated in the legend. Magenta lines correspond to the amino acid in bulk solution. Vertical lines correspond to the pI of GFP and the pKa of the acidic and basic monomers.

3.2. Adsorption from lysozyme + GFP binary mixtures

3.2.1. Polymer charge regulation

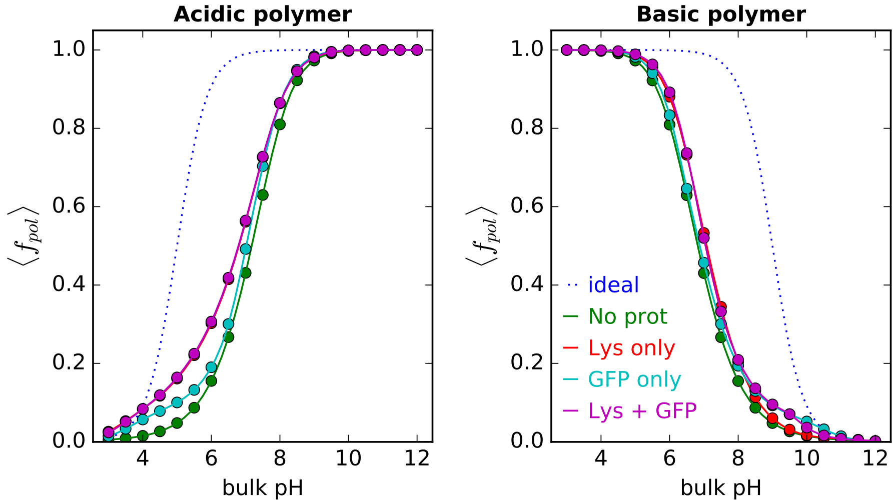

Figure S11. Average of the fraction of charged monomers as a function of bulk pH, for acidic and basic units as indicated in the header of each panel. Surface details are fixed, σ_{tot} = 0.1 nm^{-2} and x_l^{surf} = 0.5. c_{salt} = 1mM. Dotted lines correspond to the ideal titration curve of the monomer in solution. Full lines correspond to the polymer layer in contact with a solution containing no proteins, lysozyme only, GFP only, or a binary mixture of lysozyme and GFP, as indicated in the legend.

3.2.2. Local net charge of adsorbing proteins

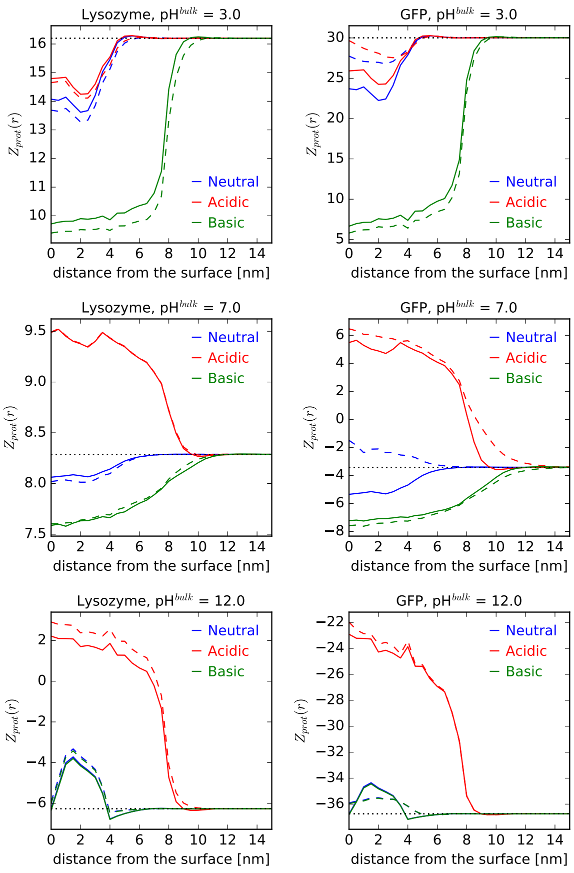

Figure S12. Local net charge of lysozyme (left panel) and GFP (right panel) adsorbed form binary mixtures in solution onto cylindrical NPs, R=5nm, as a function of the distance to the surface. Long polymers are neutral, acidic or basic, as indicated in the legend. Surface details are fixed, σ_{tot} = 0.1 nm^{-2} and x_l^{surf} = 0.5. The bulk solution conditions are c_{lys} = c_{GFP} = 10^{-4} M, c_{salt} = 1nM. The bulk pH values are indicated in the header of each panel. Full lines correspond to binary protein solutions, while dashed lines correspond to one protein solutions. The dotted vertical lines correspond to the net charge of the protein in bulk solution.

Abbreviations

The following abbreviations are used in this section:

NP	Nanoparticle
PEG	Poly(ethylene glycol)

References

1. Nap, R.J.; Tagliazucchi, M.; Gonzalez Solveyra, E.; Ren, C.l.; Uline, M.J.; Szleifer, I., Modeling of Chemical Equilibria in Polymer and Polyelectrolyte Brushes. In *Polymer and Biopolymer Brushes*; John Wiley & Sons, Ltd, 2018; chapter 6, pp. 161–221. doi:10.1002/9781119455042.ch6.
2. Nap, R.; Gong, P.; Szleifer, I. Weak polyelectrolytes tethered to surfaces: effect of geometry, acid-base equilibrium and electrical permittivity. *J. Polym. Sci., Part B: Polym. Phys.* **2006**, *44*, 2638–2662. doi:10.1002/polb.20896.
3. Carignano, M.A.; Szleifer, I. Structural and thermodynamic properties of end-grafted polymers on curved surfaces. *The Journal of Chemical Physics* **1995**, *102*, 8662–8669. doi:10.1063/1.468968.
4. Nap, R.J.; Gonzalez Solveyra, E.; Szleifer, I. The interplay of nanointerface curvature and calcium binding in weak polyelectrolyte-coated nanoparticles. *Biomaterials Science* **2018**, *6*, 1048–1058. doi:10.1039/C8BM00135A.
5. Narambuena, C.F.; Longo, G.S.; Szleifer, I. Lysozyme adsorption in pH-responsive hydrogel thin-films: the non-trivial role of acid-base equilibrium. *Soft Matter* **2015**, *11*, 6669–6679. doi:10.1039/c5sm00980d.

6. Hagemann, A.; Giussi, J.M.; Longo, G.S. Use of pH Gradients in Responsive Polymer Hydrogels for the Separation and Localization of Proteins from Binary Mixtures. *Macromolecules* **2018**, *51*, 8205–8216. doi:10.1021/acs.macromol.8b01876.
7. Hindmarsh, A.C.; Brown, P.N.; Grant, K.E.; Lee, S.L.; Serban, R.; Shumaker, D.E.; Woodward, C.S. SUNDIALS: Suite of Nonlinear and Differential/Algebraic Equation Solvers. *ACM Trans Math Software* **2005**, *31*, 363–396.
8. Flory, P.J. *Statistical Mechanics of Chain molecules*; Oxford University Press: New York, 1989.
9. Gonzalez Solveyra, E.G.; Tagliazucchi, M.; Szleifer, I. Anisotropic surface functionalization of Au nanorods driven by molecular architecture and curvature effects. *Faraday Discuss.* **2016**, *191*, 351–372. doi:10.1039/C6FD00020G.
10. Vaney, M.C.; Maignan, S.; Riès-Kautt, M.; Ducruix, A. High-Resolution Structure (1.33 Å) of a HEW Lysozyme Tetragonal Crystal Grown in the APCF Apparatus. Data and Structural Comparison with a Crystal Grown under Microgravity from SpaceHab-01 Mission. *Acta Crystallographica Section D Biological Crystallography* **1996**, *52*, 505–517. doi:10.1107/S090744499501674X.
11. Orm , M.; Cubitt, A.B.; Kallio, K.; Gross, L.A.; Tsien, R.Y.; Remington, S.J. Crystal Structure of the Aequorea victoria Green Fluorescent Protein. *Science* **1996**, *273*, 1392–1395. doi:10.1126/science.273.5280.1392.
12. Kubiak-Ossowska, K.; Jachimska, B.; Al Qaraghuli, M.; Mulheran, P.A. Protein interactions with negatively charged inorganic surfaces: simulation and experiment. *Current Opinion in Colloid and Interface Science* **2019**, *41*, 104–117. doi:10.1016/j.cocis.2019.02.001.
13. Cordeiro, A.L.; Rückel, M.; Bartels, F.; Maitz, M.F.; Renner, L.D.; Werner, C. Protein adsorption dynamics to polymer surfaces revisited—A multisystems approach. *Biointerphases* **2019**, *14*, 051005. doi:10.1116/1.5121249.
14. Patnaik, S.S.; Trohalaki, S.; Pachter, R. Molecular modeling of green fluorescent protein: Structural effects of chromophore deprotonation. *Biopolymers* **2004**, *75*, 441–452. doi:https://doi.org/10.1002/bip.20156.
15. Kleywegt, G.J.; Alwyn Jones, T. Detection, delineation, measurement and display of cavities in macromolecular structures. *Acta Crystallographica Section D: Biological Crystallography* **1994**, *50*, 178–185. doi:10.1107/S0907444993011333.
16. Grimsley, G.R.; Scholtz, J.M.; Pace, C.N. A summary of the measured pK values of the ionizable groups in folded proteins. *Protein Science* **2009**, *18*, 247–251. doi:10.1002/pro.19.
